# HIV-1 Transcripts Use IRES-Initiation under Conditions Where Cap-Dependent Translation Is Restricted by Poliovirus 2A Protease

**DOI:** 10.1371/journal.pone.0088619

**Published:** 2014-02-10

**Authors:** Raquel Amorim, Sara Mesquita Costa, Nathalia Pereira Cavaleiro, Edson Elias da Silva, Luciana Jesus da Costa

**Affiliations:** 1 Instituto de Microbiologia, Departamento de Virologia, Universidade Federal do Rio de Janeiro, Rio de Janeiro, Brazil; 2 Laboratório de Enterovírus, Instituto Oswaldo Cruz, Fundação Oswaldo Cruz, Rio de Janeiro, Brazil; Institut National de la Santé et de la Recherche Médicale, France

## Abstract

The 30 different species of mRNAs synthesized during the HIV-1 replication cycle are all capped and polyadenilated. Internal ribosome entry sites have been recognized in the 5′ untranslated region of some mRNA species of HIV-1, which would contribute to an alternative mechanism of initiation of mRNA translation. However, the Cap-dependent translation is assumed to be the main mechanism driving the initiation of HIV-1 protein synthesis. In this work, we describe a cell system in which lower to higher levels of transient expression of the poliovirus 2A protease strongly inhibited cellular Cap-dependent translation with no toxic effect to the cells during a 72-hour time frame. In this system, the synthesis of HIV-1 proteins was inhibited in a temporal dose-dependent way. Higher levels of 2A protease expression severely inhibited HIV-1 protein synthesis during the first 24 hours of infection consequently inhibiting viral production and infectivity. Intermediate to lower levels of 2A Protease expression caused the inhibition of viral protein synthesis only during the first 48 hours of viral replication. After this period both protein synthesis and viral release were recovered to the control levels. However, the infectivity of viral progeny was still partially inhibited. These results indicate that two mechanisms of mRNA translation initiation contribute to the synthesis of HIV-1 proteins; during the first 24–48 hours of viral replication HIV-1 protein synthesis is strongly dependent on Cap-initiation, while at later time points IRES-driven translation initiation is sufficient to produce high amounts of viral particles.

## Introduction

Translation in eukaryotic cells is mainly initiated via two mechanisms: one involves the recognition and association of several eukaryotic initiation factors (eIFs) to the Cap structure present at the 5′ end of all eukaryotic messenger RNAs (mRNAs); the second mechanism does not rely on the recognition of Cap but on the association of a limited number of eIFs to specific regions of highly structured 5′ untranslated regions (UTR) of mRNAs, called internal ribosome entry sites (IRES). IRES-dependent translation occurs for certain mammalian mRNAs under certain metabolic conditions [1].

Viruses must use the cellular machinery to synthesize their own proteins, as this process is highly complex and involves several components that are not encoded by the viral genomes. Moreover, especially for highly cytolytic RNA viruses, viral and host mRNAs compete for the translation machinery components. Thus, animal viruses have evolved sophisticated mechanisms to maximize the selective translation of their own mRNAs [2]. For instance, as initiation of mRNA translation is critical to ensure the synthesis of all eukaryotic proteins, and consequently is a tightly regulated step, in order to ensure synthesis of their own proteins, viruses frequently target this step [3].

The canonical translation initiation requires the recognition and binding of the mRNA through the 5′ CAP and the 3′ poly-A structures by the heterotrimeric protein complex eIF4F, which is composed of eIF4E, a protein factor that binds directly to the 5′ methyl Cap and also to eIF4A, which has RNA helicase activity. This last protein is bound by the scaffolding protein eIF4G, which by its turn further binds to the Poly-A Binding Protein (PABP), which binds the 3′ poly-A structure and approximates it to the 5′ methyl Cap. The formation of the above complex is required for the recognition and binding of the mRNA by the 43S complex, which brings both the 40S subunit of the ribosomal RNA and the tRNA-Met initiator [4].

Several viral proteins are synthesized by a non-canonical strategy of translation initiation, driven by the presence of an IRES element in a number of mRNAs. There are different types of IRES, but all contain a rich secondary structure with several stem–loops, which are responsible for this alternative ribosome recruiting pathway which does not require most of the eIFs [5]. To ensure that the translational machinery stays available only to viral mRNAs, some viruses encode proteases that cleave initiation factors, as eIF4G and PABP [6]. Under these conditions, cap-dependent and, therefore, synthesis of most cellular protein is strongly impaired, but there is no interference in IRES-driven translation [7]. One of the best-characterized viral strategies is the one promoted by members of the *Picornaviridae* family such as Poliovirus [8].

The Poliovirus genome encodes proteases that have crucial roles in the shutoff of the cellular protein synthesis. One of these proteases is named 2A protease (2APro). This small cysteine-protease cleaves the initiation factor eIF4G, which acts as a scaffold protein bridging the 5′ Cap to the 40S ribosomal subunit through eIF4E and eIF3, respectively [9]. Hence, cleavage of eIF4G by 2Apro separates the two halves of the protein and leads to an impaired Cap-dependent translation, allowing Poliovirus to turn cellular machinery to synthesize its own proteins [10].

In contrast, retroviruses such as HIV-1 perform retrotranscription and integration into a cellular genome. Once integrated, the provirus functions like any eukaryotic gene, and uses cellular factors to start its protein synthesis, which is done mainly by the Cap structure of its mRNAs [11]–[13]. However, there is still some controversy whether translation of retroviral mRNAs is strictly Cap-dependent, since some studies have demonstrated the existence of IRES elements on the 5′UTR of HIV-1, FIV and SIV [14]–[16].

In the present work, we analyzed the effect of the expression of 2APro on HIV-1 replication, investigating the importance of cap and IRES-driven translation for virus production and infectivity.

## Materials and Methods

### Plasmid Constructions

**Figure 1:**
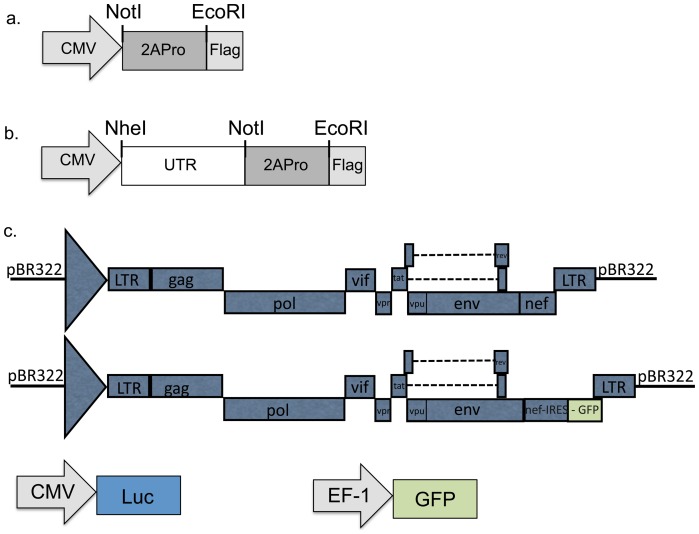
Schematic representation of plasmids used. A. HeLa cells were co-transfected with different combinations of plasmids, indicated in each experiment. A: p2A expression vector; B: pU2A expression vector; C: Expression vectors for IRES-GFP (pBR43IeG), Cap-Luciferase (pGL3) and Cap-eGFP (pTYEFeGFP).

The pCMV-2APro e pCMV-UTR-2APro expression vectors (named p2A and pU2A, respectively), were obtained by the PCR-amplification and cloning of the 5′UTR region and the 2APro gene from the Poliovirus vaccine strain Sabin-1. The 2APro gene was amplified with primers containing the NotI and EcoRI restriction endonuclease sites and was subsequently digested with the respective enzymes (2A NotI-F2 5′AAG GAA AAA AGC GGC CGC CAT GAC CAC ATA TGG ATT CGG ACA CCA A 3′/2AEcoRI-R 5′ GGA ATT CTT GTT CCA TGG CTT CTT CTT 3′). This fragment was cloned into the pCMVTag-4A vector (Stratagene, EUA) in frame with the C terminal FLAG tag to generate the p2APro (2APro under the control of the CMV promoter) vector. The Sabin-1 5′ UTR region was amplified with the following primers: 5′UTR NheI-F 5′ CTA GCT AGC TTA AAA CAG CTC TGG GGT 3′/5′UTR NotI-R 5′AAG GAA AAA AGC GGC CGC CAT TAT GAT ACA ATT GTC TGA T 3′ and introduced into the p2APro vector to generate the 2APro under the control of the CMV promoter and with an IRES-element. These constructs are depicted in Figure 1.

The HIV-1 infectious clone, pNL4-3, used in the study has been already described [17]. The bicistronic vector pBR43IeG-cpzGAB2nef is a proviral vector of HIV-1 (NL4-3 based) that expresses eGFP from a Nef-eGFP bicistronic transcript. Translation of eGFP is under the control of Encephalomyocarditis Virus (EMCV) IRES. This infectious clone was obtained through the AIDS Reagent Program, Division of AIDS, NIAID, NIH from Dr. Frank Kirchhoff [18], [19]. The GFP expression vector pTYEFeGFP was obtained through the AIDS Reagent Program, Division of AIDS, NIAID, NIH: pTY-EFeGFP from Dr. Lung-Ji Chang [20]. The Luciferase expression vector pGL3 was obtained from Promega (Promega, Madison, USA).

### Cell Culture and Transfection

HeLa cell line was obtained through the AIDS Reagent Program, Division of AIDS, NIAID, NIH, from Dr. Richard Axel and TZM-bl cell line was obtained through the AIDS Reagent Program, Division of AIDS, NIAID, NIH, from Dr. John C. Kappes, Dr. Xiaoyun Wu and Tranzyme Inc. These cell lines were grown in Dulbecco's Modified Eagle's Medium (DMEM) (Gibco, Grand Island, USA) supplemented with 10% bovine fetal serum (Gibco, Grand Island, USA), 0,23% NaHCO3 and 100 µg/mL penicillin/streptomycin (Invitrogen, Carlsbad, USA). Lymphocytic Hut78 was obtained through the AIDS Reagent Program, Division of AIDS, NIAID, NIH, from Dr. Robert Gallo and cultured in RPMI Medium 1640 (Gibco, Grand Island, USA), supplemented with 10% bovine fetal serum (Gibco, Grand Island, USA) and 100 µg/mL penicillin/streptomycin (Invitrogen, Carlsbad, USA). Cells were maintained at 37°C in a 5% CO_2_ atmosphere.

Transfection experiments were performed with 2×10^5^ HeLa cells inoculated in a 6- or 96-well plate the day before the transfection. Transfections of plasmidial DNAs were performed using Fugene 6 Transfection Reagent (Roche, Mannheim, Germany) as manufacturer recommendations. Hut78 cells were transfected using Neon™ Transfection System (Invitrogen, Carlsbad, USA), following manufacturer instructions and using three pluses of 1500 V and 10 ms. The total amount of plasmidial DNAs used were 4 µg in 6-well co-transfections, and proportions were normalized with an empty vector as follows: 1∶3: 1 µg of pGL3, pTY-EFeGFP or pNL4-3, and 3 µg of pU2A; 1∶1: 1 µg of pGL3, pTY-EFeGFP or pNL4-3, 1 µg of pU2A and 2 µg of pCDNA3.1; 3∶1: 1 µg of pGL3, pTY-EFeGFP or pNL4-3, 0,33 µg of pU2A and 2,67 µg of pCDNA3.1. For 96-well transfections, the total amount of plasmidial DNAs used was 240 ng, and proportions were normalized with an empty vector as follows: 1∶3: 60 ng of pCDNA3.1 and 180 ng of pU2A; 1∶1: 120 ng of both plasmids; and 3∶1: 180 ng of pCDNA3.1 and 60 ng of pU2A. All experiments were performed using 6-well culture plates, except of the cell viability assay. Cells were incubated for one to three days. Then the supernatants were harvested and cells were lysed by incubation with RIPA buffer (10 mM Tris-Cl pH 7.5; 5 mM de EDTA pH 8.0; 150 mM NaCl; 0.1% NP-40) for protein analysis, or fixed with formaldehyde 4% for flow cytometry.

### ELISA

P24-ELISA assays were done with the HIV-1 p24 Antigen ELISA kit (LG Lifesciences), according to the manufacturer's instructions.

### Luciferase Assay

For Luciferase assays, HeLa cells were transfected with the pGL3-control vector (Promega, Madison, USA), which encodes firefly luciferase gene. pGL3-transfected cells were lysed and processed using the Luciferase Assay System (Promega, Madison, USA) according to the manufacturer's instructions. The light emission was measured in a 96 microplate luminometer (Promega, Madison, USA).

### Detection of 2APro and HIV-1 transcripts

2×10^5^ HeLa cells were inoculated in a 6-well plate the day before the transfection. Cells were transfected with pU2A and an empty vector in a proportion of 1∶3 or with pNL4-3 and pU2A in the indicated proportions using Fugene 6 Transfection Reagent (Roche - Mannheim, Germany) according to the manufacturer's recommendations. At the indicated time, cells were processed in order to separate nuclear from cytoplasmatic portions [21]. Then, cytoplasmatic fraction was submitted to RNA extraction using the Trizol method. cDNA was generated using random primers and the High-Capacity cDNA Reverse Transcription Kits (Applied Biosystems, Foster City, USA) following manufacturer instructions. PCRs were performed using 1,25 U of Taq Platinum (Invitrogen, Carlsbad, USA) with the specific pair of primers: rRNA 18S forward: 5′ GTTAGCATGCCAGAGTCTCGTTCGTT 3′/rRNA 18S reverse: 5′ GACACGGACAGGATTGACAGATTGAT 3′/HIV-1 gagpol mRNA F: 5′ CCTCAAATCACTCTTTGGCAAC 3′/HIV-1 gagpol mRNA R: 5′ AAAATTTAAAGTGCAGCCAAT 3′; UTR-2APro: 5′UTR NheI-F 5′ CTA GCT AGC TTA AAA CAG CTC TGG GGT 3′/5′UTR NotI-R 5′AAG GAA AAA AGC GGC CGC CAT TAT GAT ACA ATT GTC TGA T 3′. The amplification of the 18SrRNA or the actin transcripts was used as loading controls. Amplification products were submitted to agarose gel electrophoresis, treatment with ethidium bromide and photographed under an UV transilluminator using a Kodak DC-40 digital system.

### Viral quantification

Viral proteins present in the supernatant were quantified by Elisa p24 assay (LG Lifesciences). The TZM-bl indicator cell line was used for the titration of HIV-1 infectious particles. Infectious blue foci were revealed by incubation with X-Gal substrate (Promega).

### Metabolic labeling of proteins

2×10^5^ HeLa cells were inoculated in a 6-well plate the day before the transfection. Cells were transfected with pU2A and an empty vector in a proportion of 1∶3 or with pNL4-3 and pU2A in the indicated proportions using Fugene 6 Transfection Reagent (Roche, Mannheim, Germany) according to the manufacturer's recommendations. At the indicated time, cells were incubated in methionine-free DMEM (Gibco, Grand Island, USA) media for 30 minutes. Then, cells were treated with 50 µM AHA (Invitrogen, Carlsbad, USA) for two hours. The cells were lysed in SDS-Tris buffer and AHA-incorporating proteins were labeled with biotin using a Click-iT Biotin Protein Analysis Detection Kit (Invitrogen, Carlsbad, USA) according to the manufacturer's recommendations. The biotin-labeled proteins were assayed by Western blotting.

### Protein analysis

Proteins were fractionated by SDS-PAGE, and transferred overnight to a nitrocellulose membrane (Hybond-ECL, GE Healthcares) on transfer buffer (20% tris-glycine 5X, 20% absolute methanol) on 20V. Primary antibodies anti-HIV-1 serum, anti-p24 and anti-eIF4G were used to detect the HIV-1 structural proteins, the HIV-1 capsid protein and cellular eIF4GI respectively. Streptavidin, horseradish peroxidase (HRP) conjugate (Invitrogen, Carlsbad, USA) was used to detect biotin-labeled proteins. HPR-conjugated second antibodies were anti-human and anti-rabbit IgGs (Santa Cruz Biotechnology. Santa Cruz, USA). Membranes were revealed by chemioluminescence using SuperSignal West Pico Chemiluminescent Substrate (Thermo Scientific, Waltham, USA) according to the manufacturer's instructions.

### *In vitro* transcription assay

*In vitro* transcription of pCMV-2APro and pCMV-UTR-2Apro plasmids was performed using the Riboprobe System – T3 (Promega, Madison, USA), following manufacturer recommendations. As the template, 1 µg/µL of each plasmid was used. As the negative control, the same amount of a pCDNA3.1 vector was used. After *in vitro* transcription, synthesized mRNAs were treated with 1U of RQ1 DNAse (Promega, Madison – USA), and finally purified using the High Pure PCR Template Kit (Roche, Mannheim, Germany).

### Polysome gradient fractionation assay

HeLa cells were transfected with pNL4-3 and pU2A in a 1∶3 proportion. After the indicated time of incubation, cells were lysed with iced polysome buffer (0,5% NP-40 + 100 µg/mL cycloheximide + 5 mM DDT (dithiothreitol) + protease inhibitor). Lysates were then passaged by a 1 mL syringe and 26-gauge needle and centrifuged at 1400 rpm at 4°C for five minutes to pellet the nuclei. Samples were submitted to a sucrose gradient from 7% to 47%, diluted in a polysome buffer (300 mM KCl, 5 mM MgCl2, 10 mM HEPES, pH 7,4) and centrifuged at 35000 rpm for 2 hours at 4°C. At the end of centrifugation, 11 aliquots of 1 mL each were collected from the top to the bottom of the gradient, and the RNA content in each fraction was extracted using the High Pure PCR Template Kit (Roche, Mannheim, Germany). cDNA and PCR were performed as described above.

### Flow cytometry

Cells transfected with pTYEFeGFP-control vector, or the bicistronic vector pBR43IeG-cpzGAB2nef, were fixed with formaldehyde 4%. The samples were analyzed in a FACScalibur cytometer, using CellQuest software version 3.1 (Becton Dickson Immunocytometry System).

## Results

### 1. Effect of 2APro expression on *de novo* protein synthesis and cellular viability

**Figure 2:**
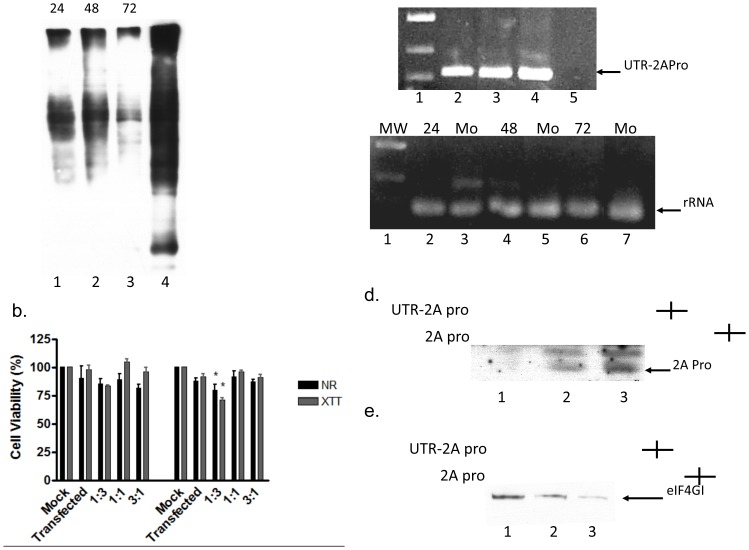
Expression of 2APro inhibits *de novo* protein synthesis without reducing cellular viability. A. HeLa cells were co-transfected with pU2A and the empty vector pCDNA 3.1 at proportion 1∶3 or mock-transfected and incubated for 24, 48 or 72 prior metabolic labeling by biotin as described. After processing, labeled proteins were submitted to SDS-PAGE followed by Western-blotting using conjugated HPR-streptavidin antibody. B. HeLa cells were transfected with different concentrations of pU2A. The total amount of DNA in each transfection was adjusted with the addition of the pcDNA3.1 empty vector. 48 or 72 hours post-transfection, cells were fixed and cellular viability was determined using XTT or neutral red dye-uptake assays. Mock cells represent non-transfected cells. Transfected represent cells that received only the transfection reagent and an empty vector. p values <0.05 were considered significant and are marked with an asterisk. C. HeLa cells were co-transfected with pU2A and the empty vector pCDNA3.1 at proportion 1∶3 or mock-transfected and incubated for 24, 48 or 72 prior RNA isolation. Polymerase chain reactions were performed using the GoTaq DNA Polymerase (Promega) and a set of primers for amplification of 2APro mRNAs or 18S rRNAs. D. HeLa cells were co-transfected with 9 µg of U2A-mRNA or control mRNA and incubated for 48 hours. After this time, cell lysates were processed by SDS-PAGE followed by western-blotting with an anti-FLAG antibody. E. HeLa cells were co-transfected with 9 µg of U2A-mRNA or control mRNA and incubated for 48 hours. After this time, cell lysates were processed by SDS-PAGE followed by western-blotting with an anti-eIF4G-I antibody.

To evaluate the effect of the expression of the Poliovirus 2APro in HeLa cells, the profile of *de novo* protein synthesis at different times post-transfection was analyzed using a metabolic labeling approach. Cells were co-transfected with pU2A and an empty vector in a proportion of 1∶3. When compared to mock transfected cells, a strong decrease in protein synthesis was observed in cells transfected with the pU2A vector at all time points (Figure 2A). This result indicates, as expected, that the 2APro is able to inhibit the *de novo* protein synthesis in HeLa cells.

In order to evaluate whether the expression of 2APro leads to negative effects in cellular viability, HeLa cells were co-transfected with different proportions of the pU2A and pCDNA3.1 plasmids (1∶3; 1∶1 and 3∶1). Cellular viability was determined after 48 or 72 hours by neutral red dye uptake and XTT assays. HeLa cells that were not transfected were used as control. During the first 48 hours, no significant differences in cell viability were observed when compared to the control, even for the highest concentration of 2APro used. At 72 hours, a slightly decrease of 20–25% in cell viability was observed with the highest concentration of 2APro expression (Figure 2B).

Several unsuccessful attempts were made to observe the levels of 2APro expression in HeLa cells by using the anti-Flag tag antibody (data not shown). Therefore, to confirm gene expression from the pU2A vector, we analyzed the levels of 2APro mRNAs transcripts present in cells up to 72 hours after transfection. We were able to detect the presence of the 2APro transcripts at all time points (Figure 2C, top panel), The amount of amplification of the constitutive cellular 18S rRNA was used as a control for the amount of total RNA present in each sample. No difference in the amount of 18S rRNA in each sample was observed (Figure 2C, bottom panel). These results strongly indicate that our construct was functional.

The pU2A vector predicts the transcription of a mRNA with a Cap-structure upstream of the poliovirus 5′ UTR (IRES). It has already been demonstrated that transcripts harboring a IRES element could lead to auto-inhibition of protein synthesis when capped [22]. Therefore, the p2A construct, which does not possess the 5′-UTR, was transfected in HeLa cells. Likewise, we were not able to detect 2APro protein levels (data not shown), indicating that the 5′-UTR region could not alone explain the undetectable levels of 2APro in our system. Other possible explanation for the undetectable levels of 2APro could be mutations in the UTR of the Sabin vaccine strain of poliovirus, which are reported to reduce poliovirus translation efficiency [23]. To test whether detectable levels of protein expression would be obtained upon transfection of 2APro mRNA, in vitro transcription of both pU2A and p2A vectors was performed. In fact, only the transfection of 9 µg of each purified transcript gave rise to detectable levels of Flag-tagged 2APro by Western-Blotting (Figure 2D). This expression correlated with the reduced levels of eIF4G-I observed in cells transfected with pU2A and p2A transcripts (Figure 2E). Importantly, the levels of UTR-2APro protein were weaker than those of 2APro (Figure 2D), confirming that the presence of a 5′-UTR region within a capped mRNA imposes partially inhibitory effects upon the expression of this protease. Nevertheless, the low levels of 2APro achieved in our system were sufficient to inhibit the *de novo* protein synthesis, but preserved cell viability for up to 72 hours of protease expression.

### 2. Effect of 2APro expression on eIF4G-I cleavage

**Figure 3:**
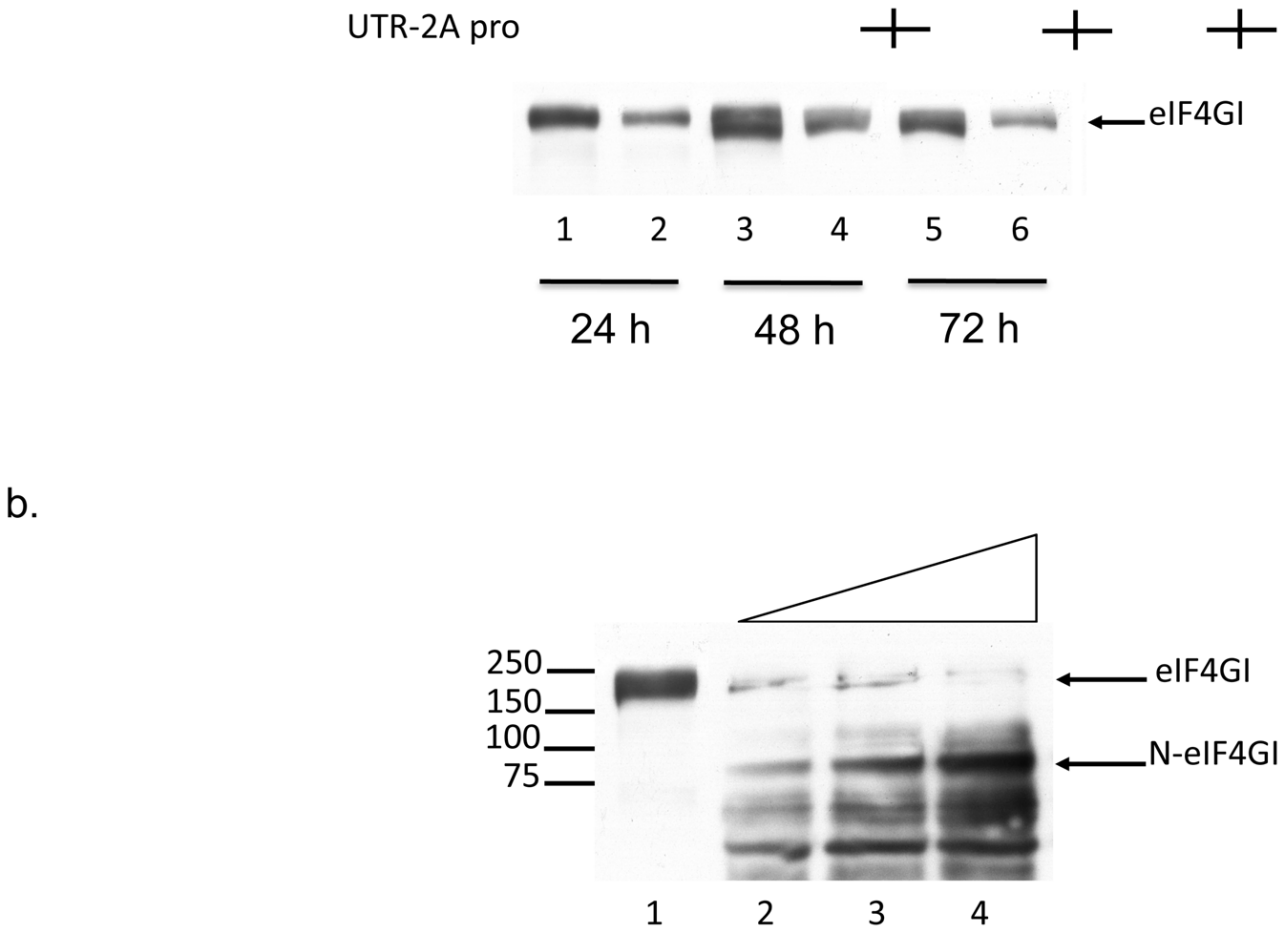
Expression of 2APro driven by pU2A construct leads to cleavage of eIF4G-I. A. HeLa cells were co-transfected with pU2A and the empty vector pCDNA3.1 at proportion 1∶3 or mock-transfected and incubated for 24, 48 or 72 hours. After this time, cell lysates were processed by SDS-PAGE followed by western-blotting with an anti-eIF4G-I antibody. B. HeLa cells were co-transfected with pU2A and the empty vector pCDNA3.1 at different proportions (1∶3, 1∶1 and 3∶1) and incubated for 48 hours. After this time, cell lysates were processed as in A.

2APro targets both isoforms of the translation initiation factor eIF4G (eIF4G-I and eIF4G-II), and this cleavage is associated with the inhibition of cellular protein synthesis [24]. Since we demonstrated that transfection of Poliovirus 2APro leads to the inhibition of *de novo* protein synthesis in HeLa cells, the presence of eIF4G-I was evaluated in these cells up to 72 hours post-transfection of 2APro. Comparing cells transfected with pU2A with mock-transfected cells, a marked decrease in the levels of non-cleaved eIF4G-I was observed from 24 to 72 hours post-transfection when the 1∶3 proportion of 2APro and the empty vector was used (Figure 3A). Co-transfections of pU2A with an empty vector at different proportions (1∶3, 1∶1 and 3∶1) demonstrated that the cleavage of eIF4G-I was strongly sensitive to the amount of pU2A present in cells, as the cleaved product N-eIF4G-I was detected in a dose-dependent way (Figure 3B). Thus, the shutoff of cellular protein synthesis correlates with the effect of 2APro upon eIF4G-I.

Usually, cleavage of eIF4G-I by Poliovirus 2APro results in the formation of a N-terminal 100kDa fragment, which could be detected with antibodies against the full-length protein. However, in our case, we were frequently unable to detect the cleavage form of eIF4G-I with the commercial antibody used in the experiments.

### 3. Effect of 2APro expression on Cap-dependent translation

**Figure 4:**
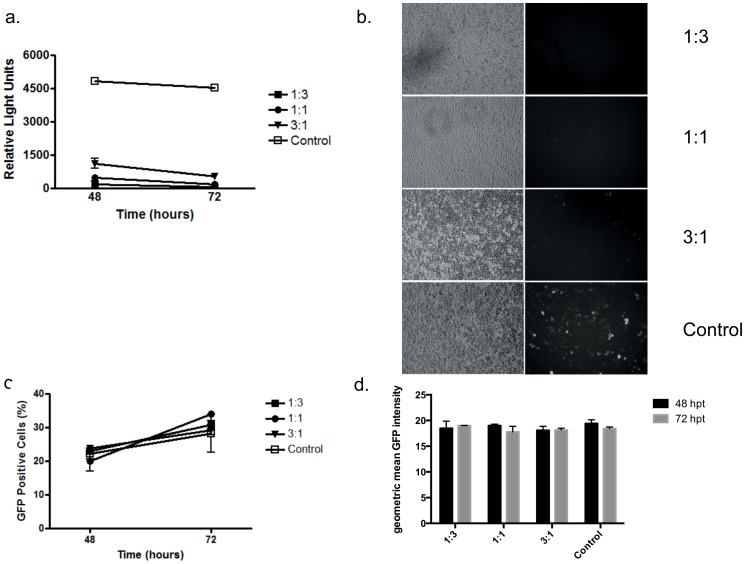
Effect of 2APro expression on protein synthesis via Cap or IRES structures. A. HeLa cells were co-transfected with the Luciferase expression vector pGL3 and pU2A in different ratios (1∶3; 1∶1; and 3∶1). The total amount of DNA in each transfection was adjusted with the addition of the pcDNA3.1 empty vector. Cells were lysed after 48 or 72 hours, Luciferase substrate was added and light was measured using a luminometer. B. HeLa cells were co-transfected with the cap-dependent eGFP expression vector and pU2A in different ratios (1∶3; 1∶1; and 3∶1). The total amount of DNA in each transfection was adjusted with the addition of the pcDNA3.1 empty vector. Cells were fixed after 72 hours of transfection and the expression of eGFP was analyzed by the emission of green fluorescence. Left column represents the bright field. C. HeLa cells were co-transfected with the HIV-1 infectious clone pBR43IeG, which express the eGFP reporter gene under the control of an IRES element and pU2A in different ratios (1∶3; 1∶1; and 3∶1). The total amount of DNA in each transfection was adjusted with the addition of the pcDNA3.1 empty vector. Cells were fixed after 48 or 72 hours and GFP expression was analyzed by flow cytometry. The percentage of eGFP positive cells was plotted. D. Geometric mean of GFP emission analyzed by flow cytometry at C. p values <0.05 were considered significant and are marked with an asterisk.

In order to evaluate the functionality of the 2APro construct on Cap-dependent protein synthesis, expression of the reporter genes Luciferase and eGFP in the presence or absence of 2APro was verified. Transcription of Luciferase and eGFP reporter genes was driven by the constitutive CMV and EF-1α promoters respectively. Different ratios of the reporter to the pU2A vector were used (1∶3; 1∶1 and 3∶1). After 48 and 72 hours, levels of protein expression were determined. As expected, Cap-dependent translation at the 1∶3 ratio was completely inhibited, as shown by Luciferase expression at 48 and 72 hours post-transfection (Figure 4A). Luciferase expression was reduced in five-fold on average when 1∶1 to 3∶1 proportions of 2Apro to the reporter gene were used, and this reduction was stable until 72 hours after transfection (Figure 4A). These results were confirmed when the eGFP reporter gene was used (Figure 4B). In contrast, no difference in levels of GFP expression in the presence and absence of 2APro was observed when an IRES-GFP reporter vector was used. No alterations in the number of eGFP positive cells (Figure 4C) or in the mean fluorescence intensity of eGFP (Figure 4D) were observed between the different concentrations of 2APro used and the mock control, confirming that the 2APro vector is functional and the shutoff of Cap-dependent protein synthesis correlates with 2APro activity.

Therefore, expression of 2Apro in HeLa cells is sufficient to inhibit Cap-dependent translation of the cellular mRNAs but does not impose a strong toxic effect on the cell culture or severely impair cell metabolism during a time period of 72 hours.

### 4. Effect of 2APro expression on HIV-1 replication

**Figure 5:**
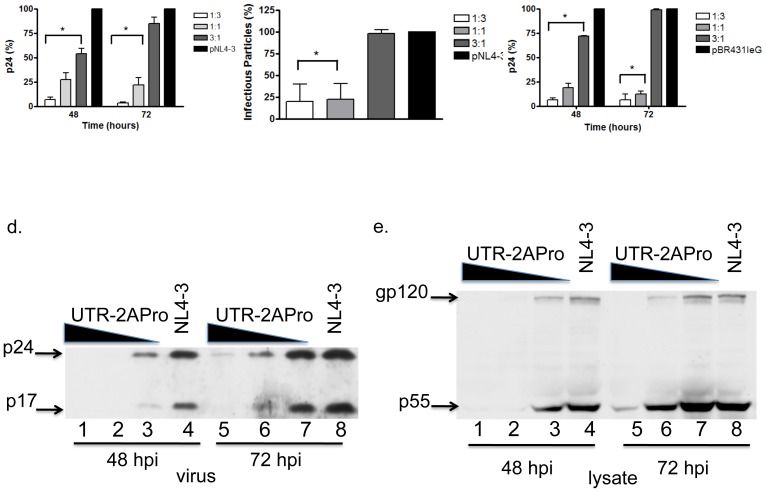
Effect of 2APro on HIV-1 replication. A. HeLa cells were co-transfected with the infectious clone pNL4-3 and the pU2A construct at different ratios (1∶3; 1∶1; and 3∶1), or pNL4-3 and the pcDNA3.1 empty vector. The total amount of DNA in each transfection was adjusted with the addition of the pcDNA3.1 empty vector. After 48 and 72 hours, virus release in the supernatant of transfected cell was detected by ELISA-p24. Standard deviations: 1∶3: 0.003536; 1∶1: 0.01662; 3∶1: 0.1842; pNL4-3: 0.2821. B. HeLa cells were co-transfected with the infectious clone pNL4-3 and the pU2A construction as in A. After 72 hours; cell supernatants were collected and used to infect TZM-bl cells. After 48 hours of infection cells were fixed and incubated with X-Gal. Blue foci were counted and plotted. Standard deviations: 1∶3: 39.98; 1∶1: 36,27; 3∶1: 8.934; pNL4-3: 0.0. C. HeLa cells were co-transfected with the infectious clone pBR43IeG and the pU2A construct as in A, and after 48 and 72 hours, virus release in the supernatant of transfected cell was detected by ELISA-p24. Standard deviations: 1∶3: 0.1821; 1∶1: 4.694; 3∶1: 18.93; pBR43IeG: 0.0. D. HeLa cells were co-transfected with the infectious clone pNL4-3 and the pU2A construction as in A. After 48 and 72 hours, cell supernatants were harvested and viral particles on the supernatants were concentrated through ultracentrifugation in a 20% sucrose cushion. Viral pellets were lyzed and analyzed for viral protein content by WB using an anti-24 antibody. Arrows indicate mature Capsid (p24) and Matrix (p17) proteins. E. HeLa cells were co-transfected with the infectious clone pNL4-3 and the pU2A construction as in A. After 48 and 72 hours, cell lysates were analyzed for HIV-1 protein content by WB using an HIV-1 anti-serum. Arrows indicate the Envelope (gp120) and Gag (p55) polyprotein precursors. p values <0.05 were considered significant and are marked with an asterisk.

In order to determine whether expression of 2APro had an effect on HIV-1 replication, co-transfections of pU2A and the pNL4-3 infectious clone of HIV-1 were made and viral protein synthesis and infectivity were determined. 2APro was able strongly to impair production of HIV-1 progeny in a dose-dependent manner. When measured by the release of mature CA-p24 in the cell-free supernatants, a tenfold- to fourfold reduction in the amount of CA-p24 was observed for the highest concentrations of 2APro used, both 48 and 72 hours after co-transfection (Figure 5A). For the lowest concentration of 2APro used (3∶1 ratio of HIV-1 to 2APro), release of CA-p24 had a twofold decrease when compared to the NL4-3 control. However, specifically at this 3∶1 ratio, amounts of CA-p24 released in the supernatant of co-transfected cells increased to the same levels as the NL4-3 control at 72 hours (Figure 5A). The amount of infectious viral particles measured at 72 hours after co-transfection confirmed the results obtained for the release of CA-p24, with 60%–75% inhibition of infectivity with 1∶3 and 1∶1 ratios of HIV-1 to 2APro, and equivalent amounts of infectivity for the 3∶1 ratio and the NL4-3 control (Figure 5B).

The same results were obtained when different concentrations of 2APro were co-transfected with the pBR43IeG infectious clone (Figure 5C), confirming that production of HIV-1 progeny is inhibited by the presence of 2APro during the first 48 hours of infection. However, synthesis of structural viral proteins is resumed in cells expressing lower levels of 2APro, leading to the release of equivalent amounts of infectious viral progeny as in the infected controls. Importantly, synthesis of the Luciferase or the eGFP reporter genes was still inhibited under these conditions (Figure 4A and B).

To further confirm these observations, we performed Western-blotting analysis of lysates and supernatants of cells co-transfected with pU2A and pNL4-3. As expected, 2APro strongly inhibited synthesis of both mature viral proteins (Ca-p24 and MA-p17) in cells supernatants and polyprotein precursors (Gag-p55 and Env-gp160) in cell lysates, at the highest proportions of 2APro (1∶3 and 1∶1) during the first 48 hours of infection (Figures 5D and E, respectively). Interestingly, synthesis of both Gag-p55 and Env-gp160 was partially recovered at 72 h of infection for the 1∶1 proportion (Figure 5E, compare lanes 6 and 8), however there was still a strong inhibition of virus production (Figure 5D compare lanes 6 and 8). The lowest proportion of 2APro used led to a modest effect on Gag-p55 and Env-gp160 synthesis (Figure 5E, compare lanes 3 and 4), but to a strong inhibition of virus production at 48 hours after infection (Figures 5D, compare lanes 3 and 4)). Moreover, a complete recovery of viral protein synthesis and viral progeny production was observed at 72 hours after infection with the lowest proportion of pU2A used (Figures 5D and E, compare lanes 7 and 8).

Collectively, this data suggests that HIV-1 translation occurs both in a Cap-dependent and an IRES-driven way. Moreover, at 48 hours of transfection, when the lowest concentration of pU2A was used, and at 72 hours of transfection, when the intermediate concentration of pU2A used, viral protein accumulated within infected cells, suggesting a release defect in these cells, which should be related to the global inhibition of Cap-dependent translation.

**Figure 6:**
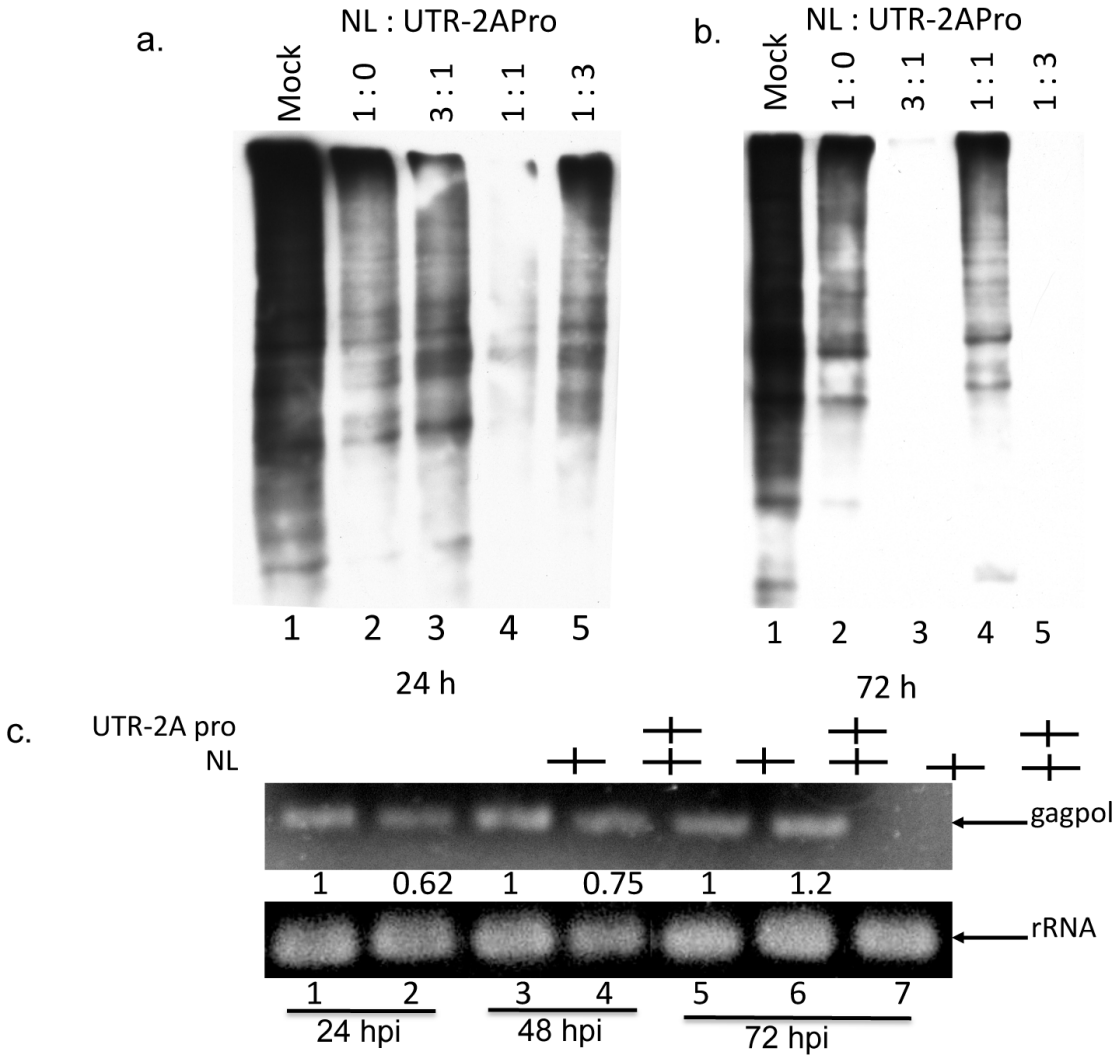
Effect of on the de novo synthesis of HIV-1 proteins and the levels of viral mRNAs. A. HeLa cells were co-transfected with pNL4-3 and pU2A at different proportions (1∶3, 1∶1 and 3∶1), with only with pNL4-3 (1:0) or mock-transfected and incubated for 24 or 72 hours prior metabolic labeling by biotin. After processing, labeled proteins were submitted to SDS-PAGE followed by Western-blotting using conjugated HPR-streptavidin antibody. B. HeLa cells were co-transfected with pNL4-3 and pU2A at 1∶3 proportion and incubated for 24, 48 or 72 hours prior RNA isolation. Polymerase chain reactions were performed using a set of primers for amplification of gagpol mRNAs or 18S rRNAs.

Importantly, metabolic labeling assays of cells co-transfected with p2APro and pNL4-3 vectors demonstrated a drastic reduction of *de novo* protein synthesis, especially at 1∶1 proportion, both at 24 or 72 hours post-transfection (Figure 6A and B, lane 4), and at the 1∶3 proportion at the 72 hours time point (Figure 6B, lane 5). Cells transfected only with pNL4-3 also had a partial inhibition of protein synthesis when compared to mock transfected cells, both at 24 and 72 hours post-transfection (Figure 6A and Figure 6B, compare lanes 1 and 2). However, it was less pronounced when compared to cells co-expressing 2APro. These results demonstrate that the recovery in HIV-1 protein synthesis at later times of 2APro expression occurred even with the maintenance of the inhibitory effect imposed on the cellular cap-depended mRNA translation.

To determine whether 2APro was interfering with HIV-1 mRNAs presence at cell cytoplasm, the levels of full-length viral mRNAs in co-transfected HeLa cells at the highest proportion of 2APro (1∶3) were analyzed. Importantly, the viral full-length mRNA was detected in cells co-expressing NL4-3 and 2APro during the entire period of the experiment. Indeed, we observed a constant increase in the viral full-length mRNA over time both for the NL4-3 alone or upon co-expression of 2APro (Figure 6C, upper panel). It will be noticed that there was a low reduction in the levels of full-length HIV-1 mRNA in 2APro co-expressing cells when compared to cells expressing NL4-3 alone. However, at 72 hours post-transfection the levels of this viral mRNAs were similar in both on cells (Figure 6C top, compare lane 5 with 6).

These results confirm that the presence of 2APro in HeLa cells infected with HIV-1, even at the highest concentration of this protease, does not interfere with the transport and therefore the presence of viral transcripts in the cell's cytoplasm.

**Figure 7:**
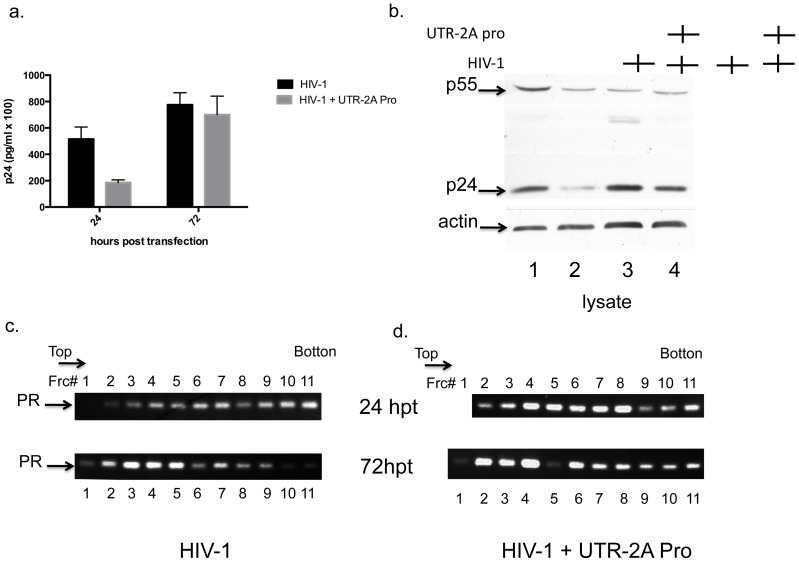
Effect of 2APro on polysome stability. HeLa cells were co-transfected with pNL4-3 and pU2A at the 1∶3 proportion or only with pNL4-3 24 or 72 hours and processed as follows: A. Supernatants were harvested and virus was detected by ELISA-p24; B Cells were lysed and presence of viral proteins was analyzed by SDS-PAGE followed by Western-blotting with anti-p24 antibody. C and D. Cells were and submitted to a polysome gradient fractionation assay, as described in methods. Fractions from top to bottom were then submitted to analysis of gagpol mRNAs associated with polysomes, in the absence (C) or presence (D) of 2APro.

To confirm the above observations, cells co-expressing NL4-3 and the lowest proportion of 2APro (3∶1) were analyzed for levels of the viral full-length mRNA associated with polysomes. Initially, analyses of cell-free and cell-associated viral proteins indicated that at 24 hours post-transfection there was three to fourfold decrease in CA-p24 levels in the presence of 2APro compared to infected cells alone (Figures 7A and 7B). However, amounts of CA-p24 in the presence of 2APro expression, both in supernatant and cell lysates, reached similar levels compared to NL4-3 alone 72 hours post-transfection, confirming previous results (Figure 5).

Analyses of viral full-length mRNA after gradient fractionation revealed that for NL4-3 alone while most of the transcripts were polysome-associate at 24 hours post-transfection (Figure 7C, upper panel: fractions 6 to 11 from the top to the bottom), at 72 hours post transfection the majority of this mRNA was associated with monosomes (Figure 7C, lower panel: fractions 1 to 5 from the top to the bottom). As expected for the presence of 2APro, most of the viral full-length mRNA was monosome-associated at 24 hours post-transfection (Figure 7D, upper panel: fractions 1 to 5 from top to bottom). This data confirms that the presence of 2APro is not inhibiting the transport of the viral transcripts to the cell cytoplasm and also indicates the functionality of 2APro in interfering with the Cap-dependent translation initiation. Interestingly, although significant levels of viral full-length mRNA are detected as monosomes at 72 hours post-transfection, a increase in polysome-associated viral transcripts is observed, especially when compared to NL4-3 alone (Figure 7D, lower panel).

### 5. Effect of 2APro on HIV-1 replication on lymphocyte cell line

**Figure 8:**
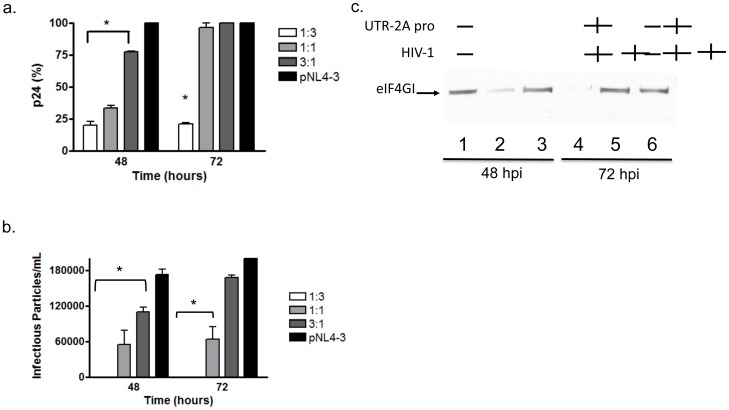
Effect of 2APro on HIV-1 replication in a lymphocytic cell line. A. Hut78 cells were co-transfected with the infectious clone pNL4-3 and the pU2A construct at different ratios (1∶3; 1∶1; and 3∶1), or pNL4-3 and the pcDNA3.1 empty vector. The total amount of DNA in each transfection was adjusted with the addition of the pcDNA3.1 empty vector. After 48 and 72 hours, virus production was detected by ELISA-p24. B. Hut78 cells were co-transfected as in A and after 48 and 72 hours, cell supernatants were collected and used to infect TZM-bl cells. After 48 hours of infection cells were fixed and incubated with X-Gal. Blue foci were counted and plotted. C. Hut78 cells were transfected with pNL4-3 (lanes 3 and 5), or co-transfected with pNL4-3 and pU2A at proportion 1∶3 (lanes 2 and 4), or mock-transfected (lanes 1 and 6) and incubated for 48 (lanes 1–3) or 72 hours (lanes 4–6). After this time, cell lysates were processed by SDS-PAGE followed by western-blotting with an anti-eIF4G-I antibody. p values <0.05 were considered significant and are marked with an asterisk.

In order to evaluate whether the inhibition of HIV-1 replication also occurs in a lymphocytic cell line, Hut78 cells were co-transfected with different ratios of pU2A and pNL4-3. As observed in HeLa cells, upon 2APro expression there was a dose-dependent inhibition of virion production from Hut78 cells during the first 48 hours, with the highest concentrations of 2APro achieving four to threefold inhibition (Figure 8A). For the highest concentration of 2APro (1∶3 ratio of HIV-1 to 2APro), inhibition of virion production was still inhibited up to 72 hours after co-transfection (Figure 8A). However, for the 1∶1 and 3∶1 ratio of HIV-1 to 2APro, virion production returned to pN4-3 control levels at 72 hours of infection (Figure 8A).

Interestingly, viral infectivity was partially inhibited for all proportions of 2APro used up to 48 hours from infection. Even when virus production was recovered at 72 hours of infection, infectivity was still inhibited for the 1∶1 proportion. Levels of infectivity similar to the pNL4-3 control were observed only at the 3∶1 condition at 72 hours after infection (Figure 8B). Finally, we also observed cleavage of eIF4GI when cells were co-transfected with pU2A at proportion 1∶3 (Figure 8C, lanes 2 and 4) compared to control cells (Figure 8C, lanes 1 and 6) or cells transfected only with NL4-3 (Figure 8C, lanes 3 and 5). The levels of 2APro transcripts were similar in all conditions (data not shown). Together, these data suggest that 2APro is also active in this cell up to 72 hours of co-expression.

The results obtained with Hut78 cell line confirm that two distinct mechanisms are involved in the translation initiation of HIV-1 mRNAs. While Cap-dependent synthesis seems to be highly required during the first 48 hours of HIV-1 replication, the IRES-driven protein translation initiation plays an important role later on the HIV-1 replication cycle. Moreover, the global inhibition of Cap-translation initiation, even without inhibiting the synthesis of viral proteins, imposes a detrimental effect on virus release and infectivity.

## Discussion

Cells infected with poliovirus present a rapid shutoff of protein synthesis, while viral mRNA translation takes place normally [25]. This phenomenon is associated with the cleavage of eIF4G, a component of the eIF4F complex, that leads to a severe impairment of Cap-dependent translation [26]. In this work, we transfected HeLa with a new 2APro expression vector, named pU2A, in order to induce an unfavorable state of Cap-dependent translation in these cells. In this condition, it is expected that IRES-driven translation occurs preferentially [27].

In cells transfected with pU2A we observed a strong shutoff in *de novo* protein synthesis until 72 hours after transfection, as expected (Figure 2A). Interestingly, a non- to mild decrease in cellular viability was observed in these conditions (Figure 2B), indicating that cells remained metabolically viable during the time frame of the experiments. It had been demonstrated that cell lines expressing 2APro suffer drastic morphological changes and die 24 hours after expression of this protease [28], and that cell death was related to the induction of apoptosis [29]. In our system neither drastic morphological changes nor cell death, measured indirectly by the XTT and neutral red assays, were observed during the 72 hours of 2APro expression (Figure 2B).

Low levels of expression of 2APro would explain this difference, since we could not detect 2APro by Western-blotting when any of our plasmid constructions (p2A or pU2A) was used in cell transfections. However, the presence of 2APro transcripts within the cell cytoplasm was confirmed (Figure 2C), as well as the cleavage of eIF4G-I in these cells (Figure 3A and 3B). These data indicate that 2APro was being expressed in these cells and was functioning as expected in inhibiting the cellular protein synthesis.

The transfection of high amounts of 2APro mRNAs from in vitro transcribed pU2A and p2A vectors allowed us to detect the Flag-Tagged 2APro. Indeed, the levels of 2APro translated from the UTR-2APro mRNA were lower than those of 2APro mRNA (Figure 2D), confirming that the presence of a 5′-UTR region upstream of a capped mRNA has negative effects upon the expression of proteins. However, this phenomenon alone could not explain the undetectable levels of this protease upon DNA transfection, since another plasmid encoding 2APro without the Poliovirus 5′-UTR also gave rise to undetectable protein levels while inhibiting Cap-dependent protein synthesis (data not shown).

The fact that protein levels of 2APro were undetectable even when high levels of 2APro transcripts were detected in the cytoplasm of pU2A-transfected cells, while detection of this protein was only possible by mRNA transfection may suggest a differential control of mRNA translation related to the presence of Cap and/or the subcellular localization of the transcripts. It has already been demonstrated that mRNA localization after transport from the nucleus influences the rate of translation and the stability of the mRNAs [30], [31].

Importantly, HeLa cells transfected with pU2A also presented expressive cleavage of eIF4G-I, up to 72 hours post-transfection (Figure 3A). Moreover, cleavage of eIF4G-I is enhanced when cells were transfected with high amounts of pU2A (Fig. 3B). We also observed a strong reduction in Cap-dependent translation from two different vectors encoding distinct reporter genes; Luciferase and GFP (Figures 4A and 4B). Therefore, despite the low levels of 2APro achieved in our transfection system, they were sufficient to inhibit Cap-dependent protein synthesis in HeLa cells.

Viral protein synthesis and consequently the production of viral particles upon 2APro expression in HeLa cells were strongly reduced. However, this effect was more drastic at higher concentrations of 2APro, in which viral replication was almost completely abolished (Figure 5). Interestingly, under conditions in which less 2APro was expressed, we observed an recovery of viral production at 72 hours of co-transfection, when compared to the HIV-1 infection in the absence of 2APro (Figure 5). Importantly, in this condition, viral protein synthesis, virion release and infectivity were re-established (Figure 5B). When equal amounts of 2APro and the NL4-3 infectious clone were transfected, the synthesis of viral polyprotein precursors within the co-transfected cells yielded amounts equivalent to the NL4-3 infectious clone expressed alone (Figure 5E). However, both viral progeny release and infectivity were still inhibited.

Several groups recently have demonstrated that the 5′-UTR of all mRNA species of HIV-1 harbors an IRES-like element [15], [32], [33]. Moreover, these IRES elements are functional in cell-free systems [34], [35]. The results of this study indicate the importance of both Cap-dependent and IRES-driven translation initiation of HIV-1 mRNAs. However, there is a time frame for its usage; for the first 48 hours of infection translation initiation of viral mRNAs is dependent on the recognition of the Cap structure. Therefore, the cleavage of eIF4G imposes a detrimental effect on viral protein synthesis. The Cap-dependence of viral mRNAS could be explained by the unavailability of host factors important for IRES recognition during the first 48 hours of expression of both 2APro and HIV-1 proteins. Nonetheless, a mechanism will exist to induce translation initiation via IRES latter on HIV-1 infection. The HIV-1 accessory protein Vpr has been implicated in the arrest of infected cells in the G2 phase of the cell cycle [36]. The G2 arrest is characterized by the dephosphorylation of eIF4E that prevents the formation of the eIF4F complex. It is reasonable to assume that Cap-dependent translation initiation of viral mRNAs becomes unfavorable and translation initiation will occur preferentially via IRES elements [15]. However, these works used bicistronic vectors to analyze the function of viral IRES [15], [37], so its functionality in the context of a viral infection it is not yet known. Nonetheless, the synthesis of HIV-1 proteins, as measured by the expression of the products of two different species of viral mRNAs (Env and Gag), was completely inhibited at 48 hours of co-transfection at the 1∶1 ratio. In this scenario, the expression of Vpr is equally unexpected.

Since the *de novo* protein synthesis in infected cells expressing different amounts of 2APro was inhibited from 72 hours post transfection (Figure 6), the recovery of viral protein synthesis at this time point for the 1∶1 and 3∶1 ratios of HIV-1 to 2APro could only be explained by the usage of the IRES element for translation initiation.

A possible effect of 2APro in the recovery of HIV-1 viral protein synthesis and production of infectious particles could be associated with polysome stability. 2APro increases the stability, translation, and replication of poliovirus mRNAs [38], by enhancing the stability of viral mRNAs and their association with polysomes [39]. We observed a different profile of association of full-length HIV-1 mRNAs with monosomes or polysomes in the presence or absence of 2APro (Figure 7, compare C and D). While at 72 hours post-transfection, HIV-1 mRNAs are normally associated with monosomes (Figure 7C), in cells that were co-transfected with 2APro, we observed an increase in association with polysomes at 72 hours (Figure 7D). This hypothesis needs to be further confirmed.

It has already been demonstrated that 2APro preferentially cleaves eIF4G-I, while the eIF4G-II isoform is more resistant to cleavage from this protease. Both eIF4G isoforms are necessary for mRNA translation initiation in eukaryotic cells. In addition, the rate-limiting step in the shutoff of host-cell translation is eIF4G-II cleavage [7], [24], [40]. eIF4G-I is more abundant in HeLa cells [41],therefore, in our system, expression of low concentrations of 2APro might lead preferentially to cleavage of eIF4G-I. Since eIF4G-I has been implicated in the pioneer round of translation initiation [42], its cleavage would affect global cell-host protein synthesis and consequently the expression of cellular factors necessary for the IRES-driven translation of viral mRNAS. Viral protein synthesis is resumed later, possibly due to the accumulation of host factors necessary to IRES recognition.

It is important to point out that the resumption of viral protein synthesis will not necessarily lead to the production of viral particles, as seen for the 1∶1 ratio of HIV-1 to 2APro in HeLa cells at 72 hours (Figure 5). In this specific case we can hypothesize that cellular factors required for successful assembly and/or release of viral progeny are not available.

Using a lymphocytic cell line as a system that is more similar to natural HIV-1 infection, this trend became even more accentuated. Complete viral replication inhibition was only reached at the highest 2APro concentrations. In contrast, when we used 2APro quantities equal to or smaller than HIV-1, viral production was similar to those encountered in the control. This probably indicates that host factors important to IRES-driven translation initiation are present early in this cell line. It is important to point out that, in this cell system, although high amounts of viral particles were produced in the presence of 2APro, they were less infectious than the NL4-3 control (Figure 8). This observation suggests that several host-cell factors, which are fundamental for modifying the cellular environment and/or the viral progeny to make it infectious are not expressed under unfavorable Cap-translation conditions. Possibly, not all HIV-1 transcripts will harbor functional IRES elements. Therefore, we cannot exclude the possibility that viral accessory proteins, such as Vif, Vpu and Nef, which contribute to HIV-1 infectivity, are not expressed.

The diverse mechanisms used by animal viruses to replicate their genomes and to translate viral mRNAs are crucial to a successful viral replication. As translation initiation is a strongly regulated step of eukaryotic protein synthesis, it is expected that viruses present strategies to subvert this machinery to benefit their own protein synthesis.

HIV-1 is strongly dependent on cellular factors for its replication [43]. However, controversy remains as to which is the prevalent pathway for its mRNAs translation: Cap or IRES-dependent. Several recent studies have proposed that translation initiation via IRES could be important to HIV-1 replication, since there are IRES-like structures located at the LTR and downstream from the gag initiation code [32], [34], [44], [45].It is possible that activation of HIV-1 IRES occurs simultaneously with G2/M cell cycle arrest, as this phase has optimal conditions to favor translation initiation driven by IRES. It has already been observed that Vpr protein or treatment with chemicals that induce G2/M arrest leads to an enhanced translation of HIV-1 mRNAs [46]–[48]. This alternative pathway of viral mRNA translation may be important to sustain HIV-1protein synthesis even in situations of global cellular translation impairment.

While this manuscript was under review, a paper came out demonstrating that both Cap-dependent and IRES-dependent translation initiation occurs at the full-length HIV-1 RNA [49]. The authors showed that either upon expression of the L-protease from FMDV or infection of HIV-1 expressing cells with Poliovirus, the 5′-UTR from the HIV-1 unspliced mRNA was active in translation initiation. These results corroborate our findings; in our study, however, we were able to demonstrate the impact of Cap-dependent inhibition not only on HIV-1 protein synthesis but also on the formation of viral infectious progeny.

Together, our data strongly suggests that HIV-1 can use both Cap and IRES translation initiation to replicate, as even in cells in which Cap-driven translation is inhibited, HIV-1 proteins and virions are detected. As already suggested in other works [34], [50], at different times of replication, HIV-1 could adopt different translation strategies, with one of the paths being preferential. Importantly, this study demonstrates that although HIV-1 could use the IRES-driven translation mechanism, it still depends on the expression of several host factors that are Cap-dependent.
